# Heredity, Health and Personal Beauty

**Published:** 1891-11

**Authors:** 


HEREDITY, HEALTH AND PERSONAL BEAUTY. By JOHN V. SHOEMAKER, A. M., M. D., Professor of Materia Medica and Pharmacology, Therapeutics and Clinical Medicine, and Clinical Professor of Diseases of the Skin in the Medico-Chirurgical College of Philadelphia ; Physician to the Medico-Chirurgical Hospital, etc. Octavo, pp. xv. —422. Philadelphia and London : F. A. Davis. 1890. Price, cloth, $2.50 ; half morocco, $3.50, net.

This is a work intended in part for the profession and in part for the laity ; that is to say,—it is written with a view to correct some popular errors regarding the rule of evolution and the law governing inheritance, and also to give the non-professional reader instruction in regard to the hygienic care of the person. While we recognize the propriety of addressing both audiences on the subjects that Dr. Shoemaker has discussed, yet we are of the opinion that it would be better to do so in separate treatises. We shall simply comment on this one with reference to the hygienic care of the person. Here readers will find how to make themselves not only healthful, but handsome in the best sense of the word. If a woman would make herself beautiful, she must first make herself healthful, because no artificial means will restore beauty or healthful color to faded cheeks, or obliterate the wrinkles that dissipation or age has produced. The chapter on Food in its relation to Health, Beauty, and Pleasure, is one that every person may read with benefit; so also in regard to that upon Clothing in its relation to Health; and Ventilation in its relation to Health, as well as the several chapters on the care of the organs of special senses. Another section of the book that is of extreme value is that comprising two chapters, relating to the Bath. There is no greater contributor to health than a properly taken bath at proper intervals. The truth is, that very few attend to bathing as a hygienic measure, and sometimes it is sadly neglected as a cleanly one. We have neither the time nor space to give this book a critical review, but commend it to the consideration of the reading public as one that will afford considerable pleasure as well as a reasonable share of benefit. The publisher has dressed the book in a neat and tasteful garb, and has printed it on excellent paper, with clear type.

